# Safety of intravenous alteplase within 4.5 hours for patients awakening with stroke symptoms

**DOI:** 10.1371/journal.pone.0197714

**Published:** 2018-05-22

**Authors:** Victor C. Urrutia, Roland Faigle, Steven R. Zeiler, Elisabeth B. Marsh, Mona Bahouth, Mario Cerdan Trevino, Jennifer Dearborn, Richard Leigh, Susan Rice, Karen Lane, Mustapha Saheed, Peter Hill, Rafael H. Llinas

**Affiliations:** 1 Department of Neurology, Johns Hopkins University School of Medicine, Baltimore, MD, United States of America; 2 Department of Emergency Medicine, Johns Hopkins University School of Medicine, Baltimore, MD, United States of America; University of Glasgow, UNITED KINGDOM

## Abstract

**Background:**

Up to 25% of acute stroke patients first note symptoms upon awakening. We hypothesized that patients awaking with stroke symptoms may be safely treated with intravenous alteplase (IV tPA) using non-contrast head CT (NCHCT), if they meet all other standard criteria.

**Methods:**

The SAfety of Intravenous thromboLytics in stroke ON awakening (SAIL ON) was a prospective, open-label, single treatment arm, pilot safety trial of standard dose IV tPA in patients who presented with stroke symptoms within 0–4.5 hours of awakening. From January 30, 2013, to September 1, 2015, twenty consecutive wakeup stroke patients selected by NCHCT were enrolled. The primary outcome was symptomatic intracerebral hemorrhage (sICH) in the first 36 hours. Secondary outcomes included NIH stroke scale (NIHSS) at 24 hours; and modified Rankin Score (mRS), NIHSS, and Barthel index at 90 days.

**Results:**

The average age was 65 years (range 47–83); 40% were women; 50% were African American. The average NIHSS was 6 (range 4–11). The average time from wake-up to IV tPA was 205 minutes (range 114–270). The average time from last known well to IV tPA was 580 minutes (range 353–876). The median mRS at 90 days was 1 (range 0–5). No patients had sICH; two of 20 (10%) had asymptomatic ICH on routine post IV tPA brain imaging.

**Conclusions:**

Administration of IV tPA was feasible and may be safe in wakeup stroke patients presenting within 4.5 hours from awakening, screened with NCHCT. An adequately powered randomized clinical trial is needed.

**Clinical trial registration:**

ClinicalTrials.gov NCT01643902.

## Introduction

Patients who wake up with acute stroke symptoms are typically excluded from treatment with intravenous alteplase (IV tPA), most commonly because these patients are outside of the standard time window. In one study, 73% of acute ischemic stroke patients were excluded from consideration for IV tPA within 3 hours, due to an elapsed time window; 24% of these patients had an uncertain time of symptom onset.[1] Patients may have an uncertain or unwitnessed time of onset due to lack of ability or a witness to communicate time of last known well (LKW), or because symptoms are discovered upon awakening. The term wakeup stroke applies to patients in whom symptoms are discovered upon awakening spontaneously.

Fink et al.[2] and Nadeau et al.[3] found that 13% to 27% of patients with acute stroke wake up with symptoms. Similarly, in the Greater Cincinnati/Northern Kentucky Stroke Study the proportion of wakeup stroke was 14%, which translates to an estimated 58,000 annual wakeup stroke patients in the US.[4] Administration of IV tPA in wakeup stroke patients, if found to be safe and effective, could result in improved outcomes of these patients, and reduce health care costs.[5]

In the Greater Cincinnati/Northern Kentucky Stroke Study, 36% of wakeup strokes would have been eligible for IV tPA if not for the time criteria, [4] confirming other studies showing that wakeup stroke patients do not otherwise differ in their tPA eligibility criteria from patients that receive tPA within 3 hours from symptom onset.[4, 6, 7]

We hypothesize that the phenotype of waking up with stroke correlates with the true time of onset and that patients presenting within 4.5 hours from waking up with stroke symptoms could be safely treated with IV tPA, if they meet all other standard criteria. In the SAfety of Intraveous thromboLytics in stroke ON awakening (SAIL ON) study, we aimed to assess proof of concept regarding safety of treatment with IV tPA in patients with acute stroke symptoms discovered upon awakening, treated within 4.5 hours from waking up and with non-contrast head CT (NCHCT) screening.

## Methods

We conducted an investigator-initiated, prospective, open label, single treatment arm, pilot safety trial of standard dose IV tPA, in patients who presented within 4.5 hours of awakening with stroke symptoms. Target enrolment was 20 patients. The study included three centers, however all patients were enrolled at either The Johns Hopkins Hospital or Johns Hopkins Bayview Medical Center. Some patients were transferred from other hospitals to these two sites for enrollment. The study was approved by the Johns Hopkins University IRB, all subjects or legaly authorized representatives gave their informed consent to participate in the trial. We obtained an Investigational New Drug (IND) from the FDA and received funds and study drug from Genentech, Inc. This study was registered in ClinicalTrials.gov, with the unique identifier NCT01643902.

From January 30, 2013, to September 1, 2015, twenty consecutive wakeup stroke patients presenting to the emergency department (ED) and selected by NCHCT were enrolled. The last patient follow up was December 1, 2015. Patients were eligible if they presented with acute stroke symptoms upon awakening, were between 18 and 80 years of age, had NIHSS ≥ 4, had a NCHCT without hemorrhage and without hypodensity of more than one third of the MCA territory, and were able to receive IV tPA within 4.5 hours from awakening. Patients with a baseline modified Rankin Scale score (mRS) of 2 or greater were excluded. Other exclusion criteria included the standard exclusion criteria for tPA administration as outlined in the 2013 AHA Guidelines.[8] All patients received IV tPA 0.9 mg/Kg, with 10% of the dose as a bolus, and the rest infused over one hour. IV tPA infusions were administered by an ED nurse or Neurological Critical Care Unit (NCCU) nurse, in the ED or NCCU. The patients in this study were not considered for endovascular mechanical thrombectomy (EMT) as they were outside of the time window for EMT based on their LKW time. Post tPA care, including the frequency of vital sign and neurological check, was according to the current standard of care; no antithrombotic therapy was administered for 24 hours. All patients were admitted to the NCCU for a minimum of 24 hours and received a NCHCT at 24 ± 6 hours post IV tPA treatment.

The primary outcome was symptomatic intracerebral hemorrhage (sICH) in the first 36 hours, as defined previously in the European Cooperative Acute Stroke Study (ECASS) 3[9] and the NINDS trial.[10] We used both definitions to increase our sensitivity in detecting sICH. The presence of intracerebral hemorrhage not meeting criteria for sICH, were deemed asymptomatic intracerebral hemorrhages (aICH). All cases with intracerebral hemorrhage as well as adverse events, were reviewed and adjudicated by an independent Safety Officer. We defined stopping rules, that were designed with the goal of ensuring safety and at the same time preventing premature ending of the trial. Given the low total “n” for the trial, it was agreed with the IRB to accept up to 20% sICH in the first half of the trial and then up to 15% sICH overall beyond the first half. The protocol defined the stopping rules as follows: during the phase of the study in which there were 1–10 subjects enrolled, the study would stop if there were two sICH. During the phase of the study in which there were 11–20 subjects enrolled, the study would stop if there were cumulatively more than three sICH. Secondary outcomes included NIHSS at 24 hours; and mRS, NIHSS, and Barthel index at 90 days.

We determined the frequencies of the primary and secondary outcomes, as well as time from last known well, and from waking up, to treatment. We also determined the time from waking up to ED arrival and the door to neele time (DNT). Stroke etiology subtype was determined using TOAST classification.[11] We also examined baseline NCHCT using the Alberta Stroke Program Early CT (ASPECT) score.

## Results

**Fig 1:**
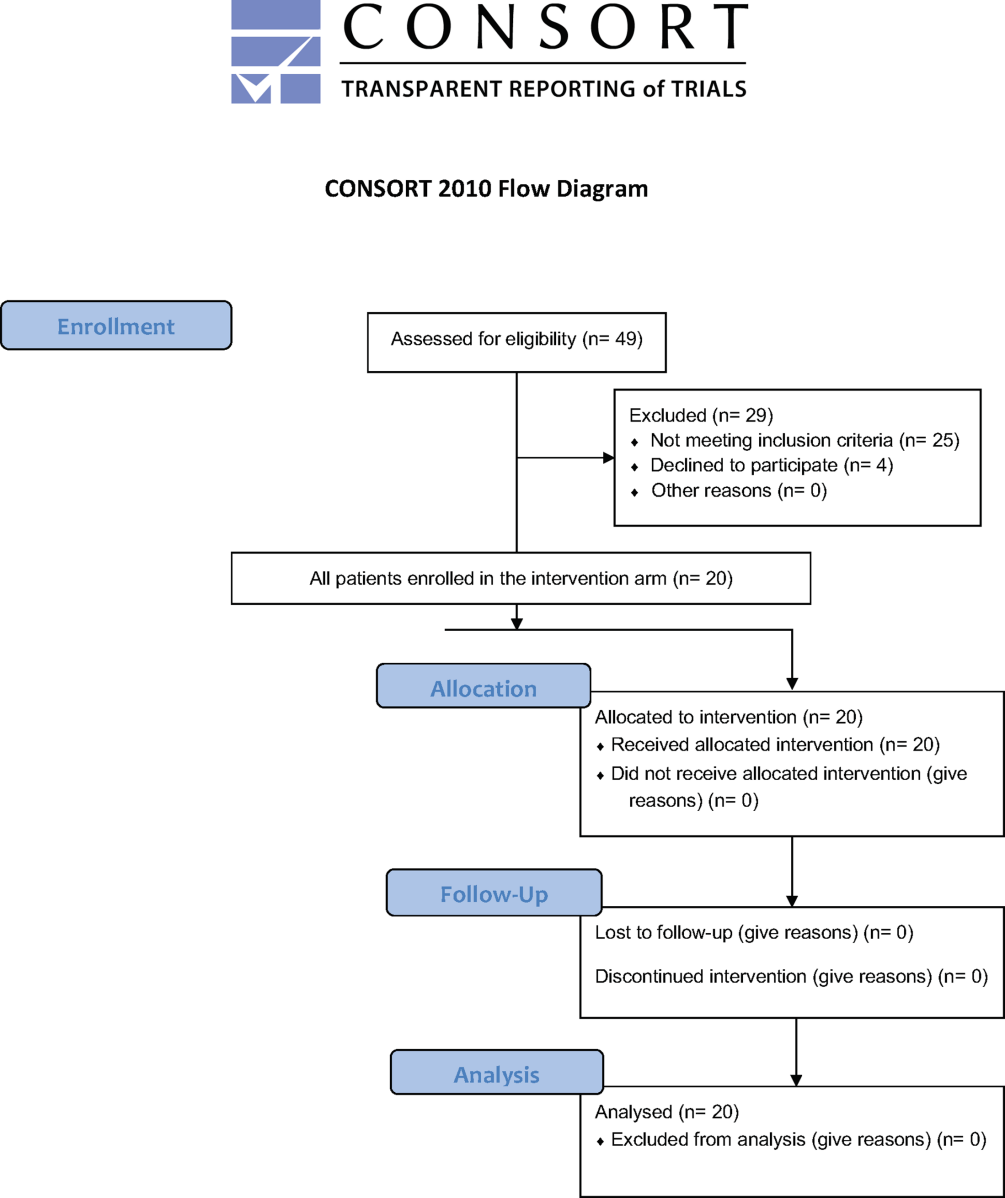
CONSORT flowchart of the SAIL ON study.

Consecutive wakeup stroke patients were screened for eligibility in the trial, a total of 49 patients were screened, 29 were excluded due to not meeting inclusion criteria or having exclusions. Four patients declined to participate and 20 were enrolled (Fig 1). One patient was enrolled that was 83 years of age, this was outside of the protocol and was reported to the IRB, corrective measures were taken, however was included in the analysis. Two patients did not have a 90 day NIHSS because their follow up visit was conducted by telephone because they were living out of the state at the time. These patients were also included in the final analysis. An intention to treat strategy was used in the analysis.

The baseline characteristics of the patients enrolled are presented in Table 1. The mean age of participants was 65 years, with a range of 47–83. Eight patients were women (40%), and 10 were African American (50%). There was one stroke mimic (5%), who was diagnosed with conversion disorder. The mean NIHSS was 6, with a range of 4–11. The mean time from awakening with stroke symptoms to treatment with IV tPA was 3 hours and 25 minutes. The mean time from last known well to treatment with IV tPA was 9 hours and 40 minutes. Mean ASPECT score for baseline NCHCT was 9.55, with a range of 4 to 10. The mean time from awakening to hospital arrival was 2 hours and 18 minutes and the mean door to needle time was 68 minutes.

**Table 1:** Baseline characteristics, n = 20.

Patient Characteristic	Value
Age, mean (range)	65 (47–83)
Female, n (%)	8/20 (40)
African American, n (%)	10/20 (50)
Last Known Well to tPA in minutes, mean (range)	580 (353–876)
Wakeup to tPA in minutes, mean (range)	205 (114–270)
Wakeup to ED arrival in minutes, mean (range)	138 (43–252)
Door to needle time in minutes, mean (range)	68 (16–177)
NIHSS, mean (range)	6 (4–11)
**Risk Factors**	
Hypertension, n (%)	13 (65)
Diabetes, n (%)	1 (5)
Atrial fibrillation, n (%)	4 (20)
Congestive heart failure, n (%)	4 (20)
Coronary artery disease, n (%)	5 (25)
Hyperlipidemia, n (%)	6 (30)
Smoking, n (%)	4 (20)
**Medications**	
Aspirin, n (%)	3 (15)
Clopidogrel, n (%)	1 (5)
Dual antiplatelet therapy, n (%)	1 (5)
Warfarin, n (%)	1 (5)
DOACs, n (%)	0 (0)
Statin, n (%)	5 (25)
**Stroke Etiology (TOAST)**	
Cardioembolic, n (%)	4 (20)
Large artery atherosclerosis, n (%)	2 (10)
Undetermined, n (%)	5 (25)
Small vessel, n (%)	8 (40)
Mimic, n (%)	1 (5)

DOAC = Direct Oral Anticoaguant

**Fig 2:**
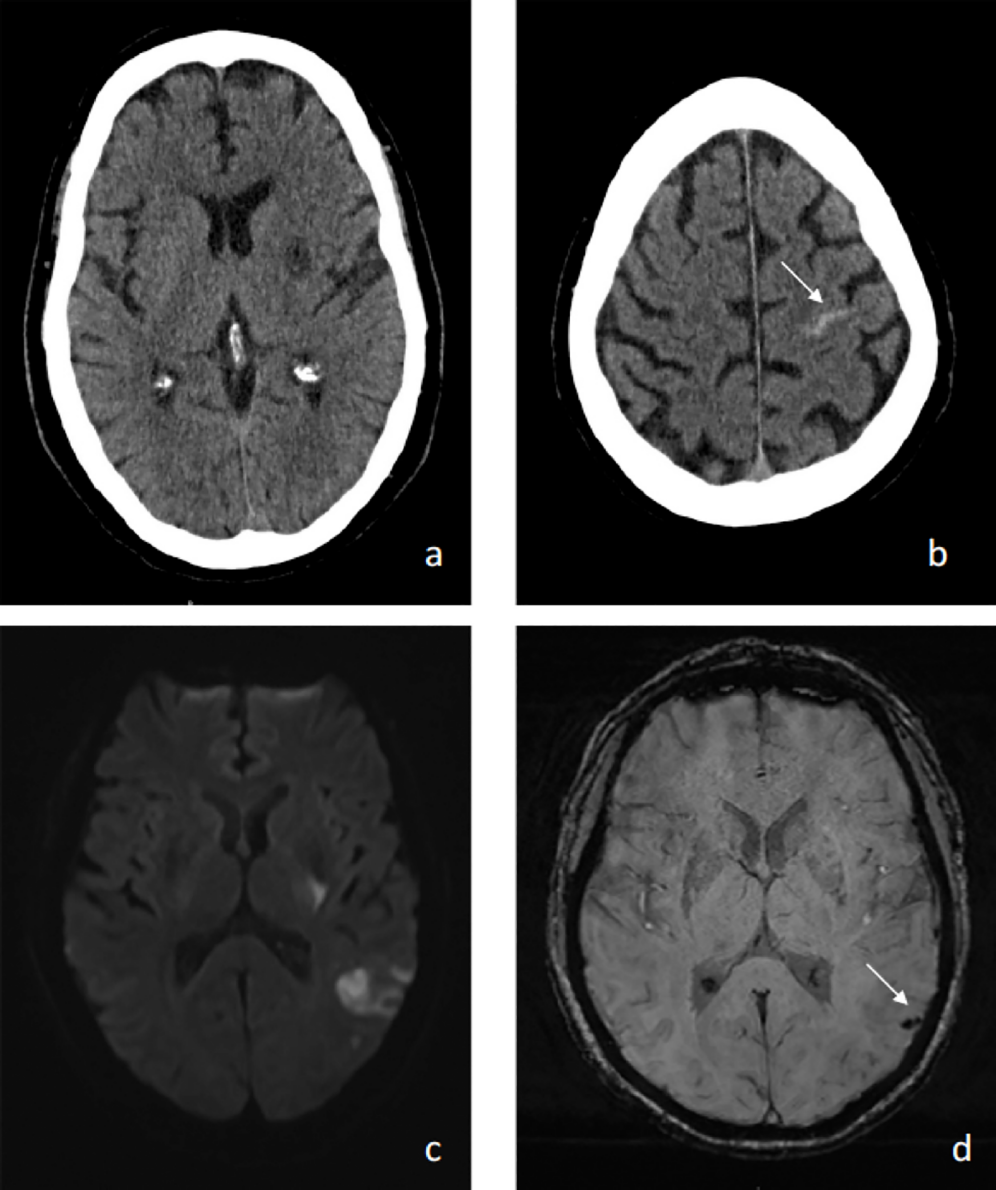
Neuroimaging is shown for the two patients with asymptomatic hemorrhage. Images (a) and (b) are from the 24 hour NCHCT on subject #2, images (c) and (d) are from the follow up MRI on subject #12. The arrows point to areas of hemorrhage.

There were no symptomatic intracerebral hemorrhages (95% CI, 0–19%).[12] There were only two asymptomatic intracerebral hemorrhages (Table 2). One of the aICH was discovered incidentally on MRI, the other on 24 hour follow up CT (Fig 2). All patients completed follow up with mRS and Barthel index at 90 days. Two patients did not complete NIHSS at 90 days. The median mRS at 90 days was 1 (range 0–5), 70% had a mRS of 0–1, mRS of 2, 3, and 5 were 10% each at 90 days. Mean Barthel index at 90 days was 18.25, with a range of 1 to 20. Mean NIHSS at 90 day follow up was 1.6. There were no deaths by 90 days (Table 2).

**Table 2:** Primary and secondary outcomes.

Outcome	Value
sICH n (%)	0 (0)
aICH n (%)	2 (10)
Median mRS at 90 days (range)	1 (0–5)
Mean Barthel index at 90 days (range)	18 (1–20)
Mean NIHSS at 90 days (range)	1.6 (0–16)

Based on TOAST classification,[11] 4 (20%) strokes were cardioembolic, 5 (25%) were of undetermined etiology, 8 (40%) were small vessel stroke, 2 (10%) were large artery stroke, and 1 (5%) was a stroke mimic. The two strokes caused by large artery atherosclerosis had NIHSS of 10 and 11. One of them had an aICH, this subject that presented with an artery to artery embolus from a extracranial internal carotid stenosis, made a complete recovery after recanalizing the previously occluded middle cerebral artery. The other presented with a complete occlusion of the extracranial internal carotid artery and did not recanalize. This was one of two subjects with a 90 day mRS of 5. The second aICH was in a cardioembolic stroke with baseline NIHSS of 7, also with complete recovery. Regarding severity; 8 (40%) of the cohort had a baseline NIHSS ≥ 7, and 4 (20%) had a baseline NIHSS ≥ 10.

## Discussion

The results of this sudy provide proof of concept that patients with wakeup stroke, presenting within 4.5 hours from waking up, can be treated with IV tPA using NCHCT for selection and following the standard IV tPA treatment protocol. Given that this trial is a pilot with 20 patients, we cannot provide information on potential efficacy. Subjects enrolled in this trial had a mean NIHSS at baseline of 6, which may have an impact in the functional outcome results, which demonstrate 70% mRS of 0–1. While in the European Cooperative Acute Stroke Study (ECASS) 3, the mean NIHSS was 11 (median 9.5), a recently published wakeup stroke study reported a median NIHSS of 6.5.[9, 13] The low NIHSS may also be related to the lack of sICH observed in the study, however given the low “n”, the results serve as proof of concept and therefore the true sICH frequency may be higher or lower. In addition, the mean NIHSS in our study is not unusual compared to reported studies of IV tPA in wakeup or unwitnessed stroke patients.[14, 15] In light of this, our results are intriguing, particularly in the context of the relatively long time from LKW to treatment with IV tPA.

These findings are consistent with other studies evaluating the safety of IV tPA in wakeup stroke patients which revealed no excess in sICH; this study is particularly significant because of the treatment time window used (4.5 hours from awakening) and the use of NCHCT-only patient selection.[6, 13, 15–17] A recent trial of wakeup stroke patients treated with tPA, suggested safety of patient selection by NCHCT. The Wake-Up Stroke study[13] included prospectively enrolled patients with wakeup stroke and treated with IV tPA within 3 hours from waking up and selected by non-contrast head CT (NCHCT). The ability to safely treat patients with wakeup stroke using IV tPA based on NCHCT alone is significant as this imaging modality is widely available. Our pilot trial was designed to be pragmatic and reflect current practice, therefore increasing generalizability. We chose the time window of 4.5 hours because the practice of treating stroke patients with IV tPA up to 4.5 hours from time of LKW is widespread in the United States, based on the ECASS 3 trial and the 2009 AHA Science Advisory.[9, 18]

As of 2015, EMT has been demonstrated to be efficacious up to 6 hours, as reflected in the current AHA guidelines.[19] Close analysis of the IV tPA and EMT trials reveals an outcome-time relationship that slopes down to lesser odds of recovery as time to treatment increases, with a time limit between 4.5–6 and 7.5 hours for IV tPA, and EMT respectively.[20, 21]

The most common reason for not qualifying for acute revascularization therapy (with IV tPA or EMT) is being outside of the established treatment windows; in fact, a 2011 study of hospitals participating in the Get With The Guidelines registry revealed that only 7.0% of stroke patients are treated with IV tPA.[22]

Most patients (64%) present beyond 6 hours from LKW. Among these patients, unknown time of onset and wakeup strokes, constitute an important proportion. [23] Further, three recent clinical trials of unwitnessed stroke report that the majority of unwitnessed stroke patients enrolled had their symptoms discovered upon awakening. In MR WITNESS, WAKE UP, and DAWN, the proportion of wakeup strokes was 71%, 85%, and 64.5% respectively.[14, 15, 24] These studies used advanced imaging, either MRI or CT perfusion (CTP) to determine elegibility based on two different paradigms. MR WITNESS and WAKE UP used an MRI surrogate of time of onset: the DWI/FLAIR mismatch; DAWN used an imaging/clinical correlate of salvageable tissue vs. core infarct: NIHSS and MRI (DWI) or CTP based relative cerebral blood flow (rCBF) volume. This may limit the generalizability, as advanced imaging is not as widely available as NCHCT. A 2005 survey of hospitals with Emergency Departments found that 96% of hospitals had a CT scanner available, but only 13% had an MRI scanner and a 24/7 technologist on site; an additional 26% had MRI capability with 24/7 on-call technologists.[25]

Additionally, the results of the DAWN and DEFUSE 3 trials are only applicable to large artery occlusion (LVO) patients. As reported in the DAWN paper, based on restrospective studies, a third of patients with LVO in the anterior circulation may meet the trial’s eligibility criteria.[24, 26] Therefore, the majority of patients might not have LVO and could benefit from IV tPA. While EMT is effective for LVO, patients with small branch occlusion and small vessel disease could still benefit from IV tPA, and will not qualify for EMT. Thus, wakeup stroke patients presenting within 4.5 hours from waking up, could be treated with standard protocols for IV tPA and EMT, without a requirement for advanced imaging criteria. This would produce the greatest expansion of the population eligible for acute reperfusion treatment, as patients with any degree of severity with or without LVO could qualify for treatment. Moreover, because they could be selected by NCHCT, which is widely available, treatment protocols could be implemented in all primary stroke centers. Patients with suspected LVO can be further evaluated with CT angiography or MRI where available, to determine elegibility per DAWN or DEFUSE 3.

### Limitations

Several limitations are noted in this study. First, the small sample size did not allow for further exploration of efficacy; however, the intent was not to definitively demonstrate efficacy, but to provide safety data in order to develop a large definitive clinical trial. Other limitations include the single arm and open label design. We did not keep screening logs, however, all patients meeting inclusion/exclusion criteria who were approached for enrollment agreed to participate and were entered into this study.

### Next steps

An adequately powered randomized clinical trial is needed to confirm safety and demonstrate efficacy of IV tPA for wakeup strokes within 4.5 hours of awakening. Our results serve as proof of concept that NCHCT as a screening tool should be considered in a future trial aiming to demonstrate the safety and efficacy of IV tPA in wakeup stroke patients. Use of NCHCT to identify eligible patients will allow for greater generalizability in clinical practice, should the administration of IV tPA in wakeup strokes within 4.5 hours of awakening be proven safe and effective.

## Conclusion

The SAIL ON study is a prospective clinical trial of wakeup stroke treatment with IV tPA, using a 4.5 hours time window from waking up with symptoms, and selected with NCHCT. Administration of IV tPA in wake-up stroke patients was feasible and may be safe. An adequately powered randomized clinical trial is needed to confirm safety and efficacy.

## Supporting information

S1 File Trendstatement_TREND_Checklist.SAIL ON. (PDF)

S2 File Supporting Information.anonymized.data. (XLSX)

S3 File Urrutia-Protocol_SAIL ON.V6. (DOC)
