# Supplementary material for: Safety of intravenous alteplase within 4.5 hours for patients awakening with stroke symptoms
Source: PLoS One. 2018 May 22;13(5):e0197714. doi: 10.1371/journal.pone.0197714 (PMC5963768; doi:10.1371/journal.pone.0197714)
Supplement: S3 File — (DOC) [file pone.0197714.s003.doc]

# PROTOCOL

**Study Title: SA**Fety of **i**ntravenous thrombo**l**ytics in stroke **on** awakening **(sail-on) Phase II trial**

**Study Drug: Activase ® (Alteplase, recombinant)**

**IND:**

**Sponsor : Johns Hopkins University**

**school of medicine**

**601 N Caroline St, suite 5073A**

**Baltimore, MD 21287**

**Investigator : Victor C. urrutia, MD**

**Sub-Investigators:**

**Date Final: 09/02/2012**

TABLE OF CONTENTS

Page

1. introduction 6

1.1 Activase Background 7

1.1.1 Drug Biochemistry and Formulation 7

- - 1. Indications 8

2. OBJECTIVES 9

2.1 Efficacy Endpoints 9

2.2 Secondary Endpoints 9

3. study Design 9

3.1 Description of the Study 9

3.2 Rationale for Study Design 11

3.3 Outcome Measures 11

3.3.1 Primary Efficacy Outcome Measures 11

- - 1. Secondary Outcome Measures 12
    2. Safety Outcome Measures
  1. Safety Plan 12
  2. Compliance with Laws and Regulations 12

4. Materials and methods 12

4.1 Subjects 13

4.1.1 Subject Selection 13

4.1.2 Inclusion Criteria 13

4.1.3 Exclusion Criteria 14

4.2 Method of Treatment Assignment 16

4.3 Study Treatment 16

4.3.1 Formulation 16

4.3.2 Dosage, Administration, and Storage 16

4.3.3 Dosage Modifications 17

4.4 Concomitant and Excluded Therapy 17

4.5 Study Assessments 17

4.5.1 Screening and Pretreatment Assessments 1/P

4.5.2 Assessments during Treatment 1/P

4.5.3 Follow‑Up Assessment 2/P

4.6 Subject Discontinuation 2/P

- 1. Study Discontinuation 2/P

4. Materials and methods (cont’d)

4.8 Statistical Methods 2/P

4.8.1 Analysis of the Conduct of the Study 3/P

4.8.2 Baseline Demographics 3/P

4.8.3 Safety Analysis 3/P

4.8.4 Efficacy Analysis 3/P

4.9 Data Quality Assurance 4/P

Assessment of safety 4/P

5.1 Specification of Safety Variables 4/P

5.1.1 Adverse Events 4/P

5.1.2 Serious Adverse Events 6/P

5.2 Methods and Timing for Assessing and Recording Safety Variables 6/P

5.2.1 Adverse Event Reporting Period 6/P

5.2.2 Assessment of Adverse Events 6/P

5.3 Procedures for Eliciting, Recording and Reporting Adverse Events 7/P

5.3.1 Eliciting Adverse Events 7/P

5.3.2 Specific Instructions for Recording Adverse Events 8/P

5.4 Additional Reporting Requirements for IND Holders 10/P

5.5 Study Close Out……………………………………………………… 13/P

6. investigator requirements 13/P

6.1 Study Initiation 13/P

6.2 Study Completion 14/P

6.3 Informed Consent 14/P

6.4 Institutional Review Board Approval 15/P

6.5 Case Report Forms 16/P

6.6 Study Drug Accountability 16/P

6.7 Disclosure of Data 16/P

6.8 Retention of Records 17/P

TABLE OF CONTENTS (cont'd)

Page

REFERENCES……………………………………………………………………. 18/P

APPENDICES

Appendix A: Study Flowchart (s)………………………………………………… 20/P

Appendix B: Safety Reporting Fax Cover Sheet……………………………………….. 21/P

Appendix C: Analysis of Similar Event………………………………………….. 22/P

Appendix D: Informed Consent…………………………………………………..

1.0 INTRODUCTION

Justification and Rationale for this Trial

Intravenous ACTIVASE (Alteplase, recombinant) is the only FDA-approved therapy for acute stroke. Yet, a narrow time window limits its use; treatment must be started within three hours of symptom onset. While this time window has been established based on pre clinical work and supported by the pooled data analysis of all the IV rt-PA trials (The ATLANTIS, ECASS, and NINDS rt-PA Study Group Investigators Lancet 2004) which show a decreasing benefit as the time from onset becomes longer, it is the major hurdle for treating patients with acute stroke with IV rt-PA.

The recent publication of the ECASS 3 trial (Hacke W, et al, NEJM 2008) has opened the window for selected patients to receive IV rt-PA for acute stroke up to 4.5 hours from onset. While this is a great improvement, it still requires knowledge of the time of onset or the time of last seen well, which excludes a majority of patients that wake up with a stroke for thrombolytic therapy. Patients who wake up with acute stroke symptoms have, to date, been excluded from the only approved treatment for acute stroke and most trials of reperfusion therapies, except the ABESTT-II trial, which randomized patients with wake up stroke to abxicimab vs. placebo (21 vs. 20 respectively). The wake up arm of this study was stopped after 3 of the 21 (14%) treated patients suffered a symptomatic intracerebral hemorrhage compared with one in the placebo group. (Adams, et al, Stroke 2008)

Intravenous rt-PA (IV tPA) continues to be underutilized as only 1.12% of patients admitted for acute ischemic stroke between 1999-2004 received this treatment (Schumach*er et* al, 2007). Surprisingly, in academic medical centers, the rate is only slightly higher: 4.1% (Johnst*on et* al, 2001). The most common reason that patients are deemed ineligible for IV rt-PA therapy is that they present outside the three-hour time window. In one study, 73% of acute ischemic stroke patients were excluded from consideration for IV rt-PA therapy due to an elapsed time window. Within this group, 24% of patients had an uncertain time of symptom onset (Barb*er et* al, 2001). Fink et al (Stroke 2002) and Nadeau et al (Can J Neurol Sci 2005) found that 13% to 27% of patients with acute stroke wake up with symptoms.

There is evidence that suggests the time of onset of most “wake up” strokes is upon awakening. If this is the case, treating patients that wake up with stroke and present to the Emergency Department (ED) within 3 or 4.5 hours of onset may be feasible and safe. A summary of relevant studies is below under “Supporting Information and Prior Clinical Experience”.

We propose to test whether treatment with IV rt-PA is safe in patients with acute stroke symptoms upon awakening. If successful, it will be the first step in planning for a confirmatory study that will determine if there is an improvement in outcomes with IV rt-PA vs the current standard. A successful confirmatory trial will open treatment with IV rt-PA to this population of patients that represents between 13 to 27% of all acute stroke patients.

Aside from improving the outcome of these patients, which is in itself an important goal, it is projected that in an integrated health system (Hospital, rehabilitation, nursing home) there is a saving of $7 Million per year per every 2% increase in rt-PA use. (Deamaerschalk et al, Stroke 2005).

Supporting Information and Prior Clinical Experience

Kelly-Hayes et al (Stroke 1995) reported data from the Framingham study suggesting that strokes are more than twice as likely to occur between 4 and 8 am, than in the previous four hours from midnight to 4 am. This suggests that many strokes that are discovered upon awakening have occurred in the early morning hours just prior to awakening. Todo and collaborators (Cerebrovasc Dis, 2006) compared CT findings in patients with acute stroke. They evaluated three groups: Patients with known time of onset within 3 hours, patients with stroke symptoms present upon awakening, and patients with unknown time of onset. They found that hypodensity in the CT was present most often in the group of patients without a known time of onset than in the other two groups, suggesting a similarity between patients waking up with stroke and patients presenting within 3 hours of onset, which could be interpreted as patients waking up with stroke symptoms having an onset of stroke within the 3 hour window.

Barreto et al (Stroke 2009) conducted a retrospective analysis of patients with wake up stroke treated with thrombolytic therapy. These patients had no significant excess symptomatic intracerebral hemorrhage and showed improved functional outcomes compared with patients with wake up stroke that were not treated with rt-PA in their case series. The mortality however was higher in the rt-PA treated group, this was interpreted as being an effect of selection bias favoring more severe strokes to receive thrombolytic therapy and the cause of death was related to failed recanalization rather than symptomatic intracerebral hemorrhage.

Potential Risk to Human Subjects

The main potential risk of testing IV rt-PA in patients with wake up stroke is symptomatic intracerebral hemorrhage. The goal of this learning phase study is to determine safety. If shown to be safe in terms of symptomatic intracerebral hemorrhage rate, a confirmatory trial will be done to demonstrate efficacy.

1.1 Activase Background

1.1.1 Drug Biochemistry and Formulation

Tissue Plasminogen Activator as found in tissue or in the melanoma cell line is a serine protease glycoprotein varying in molecular mass from 63,000 to 65,000 daltons. The molecular mass variation reflects heterogeneity due to different patterns of glycosylation. It contains an amino‑terminal region that has a high degree of sequence homology with the “kringle” regions of plasminogen (Sottrup‑Jensen et al. 1978) and prothrombin. A kringle is a characteristic triple disulfide structure originally described in the “pro” fragment of prothrombin (Magnusson et al. 1975). The amino‑terminal region may be responsible for the fibrin‑specific activation of rt‑PA. The carboxy‑terminal end of the molecule contains a domain responsible for the protease activity of rt‑PA. rt‑PA is a mixture of one‑chain and two‑chain forms. The composition depends on the amount of proteolysis that takes place during manufacturing.

Activase® (Alteplase, recombinant; recombinant tissue plasminogen activator, rt‑PA) is commercially available as a lyophilized powder for reconstitution in 100- and 50-mg vials.

Activase [Alteplase] is a tissue plasminogen activator (rt‑PA) produced by recombinant DNA technology. It is a sterile, purified glycoprotein of 527 amino acids. It is synthesized using the complementary DNA (cDNA) for natural human tissue‑type plasminogen activator (rt‑PA) obtained from an established human cell line. The manufacturing process involves secretion of the enzyme Alteplase into the culture medium by an established mammalian cell line (Chinese hamster ovary cells) into which the cDNA for Alteplase has been genetically inserted. Fermentation is carried out in a nutrient medium containing the antibiotic gentamicin sulfate, 100 mg/L. The presence of the antibiotic is not detectable in the final product.

Activase is a sterile, white to pale yellow, lyophilized powder for intracatheter administration (instillation) for restoration of function to central venous access devices following reconstitution with Sterile Water for Injection, USP.

Activase is commercially available as a lyophilized powder for reconstitution 2-mg vials.

1.1.2 Indications

a. Acute Myocardial Infarction

Activase is indicated for use in the management of AMI in adults for the improvement of ventricular function following AMI, the reduction of the incidence of congestive heart failure, and the reduction of mortality associated with AMI. Treatment should be initiated as soon as possible after the onset of AMI symptoms.

b. Acute Ischemic Stroke

Activase is indicated for the management of acute ischemic stroke in adults for improving neurologic recovery and reducing the incidence of disability. Treatment should only be initiated within 3 hours after the onset of stroke symptoms and after exclusion of ICH by a cranial CT scan or other diagnostic imaging method sensitive for the presence of hemorrhage.

c. Pulmonary Embolism

Activase is indicated in the management of acute massive PE in adults:

- For the lysis of acute pulmonary emboli, defined as obstruction of blood flow to a lobe or multiple segments of the lungs
- For the lysis of pulmonary emboli accompanied by unstable hemodynamics (e.g., failure to maintain blood pressure without supportive measures)

The diagnosis should be confirmed by objective means, such as pulmonary angiography or noninvasive procedures such as lung scanning.

2.0 OBJECTIVES

The primary objective of this study is to evaluate the safety of intravenous rt-PA in patients waking up with symptoms of acute stroke and presenting to the ED within 4.5 hours from awakening and having either a non-contrast head CT without hemorrhage and without hypodensity more than 1/3 of the MCA territory; or MRI demonstrating no hemorrhage, and with a DWI lesion no greater than 70mL and FLAIR without a well demarcated hyperintense lesion that is more than 1/3 of the MCA territory.

Phase II, safety study.

2.1 primary Endpoints

1. Symptomatic intracerebral hemorrhage (sICH) within 36 hours of treatment. We will collect data on symptomatic intracerebral hemorrhage by using the ECASS 3 criteria as well as the original NINDS IV rt-PA trial criteria for comparison.

- In ECASS 3 symptomatic intracerebral hemorrhage is defined as: Any extravascular blood in the brain or within the cranium associated with a clinical deterioration defined as an increase in 4 points on the NIHSS, or that lead to death and was identified as the predominant cause of neurological deterioration.
- In the original NINDS IV rt-PA trial symptomatic intracerebral hemorrhage is defined as: A hemorrhage not seen in previous CT scans and there was a suspicion of hemorrhage or any deterioration in neurologic status.

2.2 secondary Endpoints

1. Asymptomatic intracerebral hemorrhage occurring at any time during the study.
2. Symptomatic intracerebral hemorrhage by ECASS 3 and the original NINDS IV rt-PA trial criteria occurring at any time during the study.

3. Efficacy measures:

a. Modified Rankin score, NIHSS, Barthel index at 90 days

b. NIHSS at 24hrs

c. Mortality at 90 days.

- 1. STUDY DESIGN
  2. **DESCRIPTION OF THE STUDY**

Design:

The study proposed is a one arm, open label study. The aim is to test the safety of IV rt-PA in stroke upon awakening.

The null hypothesis (Ho) is that IV rt-PA in this population will have a symptomatic intracerebral hemorrhage rate of less than 6%.

The alternative hypothesis (Ha) is that IV rt-PA will have a symptomatic intracerebral hemorrhage rate of more than 10%, which would be unacceptably high.

Treatment Regimen:

IV rt-PA 0.9mg/kg, maximum of 90mg. Administered by the standard protocol: 10% of the dose by Intravenous bolus injection, followed by infusion of the remainder over 1 hour.

Treatment will be initiated within 4.5 hours of awakening, with a preferred target door to needle time of 60 minutes or less from arrival to the ED (i.e. the 60 minute door to needle time is a goal but not required for enrollment since the national target for standard IV rt-PA door to needle time is less than 60 minutes in 50% of patients treated).

No antithrombotic medications in the first 24 hours. After that period, choice of antithrombotic medication will be at the discretion of the treating physician

Study Interventions:

Once the patient has agreed to participate and has signed the consent form, a subject ID number will be assigned. Prior to be enrolled in the study, all subjects will have received the following procedures/tests:

1. History and physical examination.
2. EKG, CBC, electrolytes, creatinine and BUN, PT and PTT.
3. Two intravenous catheters will be started.
4. A non-contrast CT scan of the brain or MRI of the brain per the standard of care protocol at the clinical site.

The subject will then receive open label rt-PA via IV catheter at a dose of 0.9mg/kg, 10% given as a bolus and the remainder given as a continuous infusion over one hour. The maximum dose is 90mg.

The study subject will be monitored in the ED until the completion of the rt-PA infusion, following the standard protocol for monitoring after rt-PA administration for acute stroke within 4.5 hours of onset. Once the infusion is completed, the subject will be transferred to the intensive care unit where the observation as per the standard clinical care protocol will be followed. Blood pressure and neurological status will be closely monitored as per protocol. Observation in the intensive care unit will be for a minimum of 24 hours. Subjects will be under the care of the admitting physician that may also be the study investigator.

A non-contrast head CT will be done between 20-30 hours after administration of rt-PA and at any time before or after if there is the clinical suspicion of hemorrhagic transformation or a decline in the subject’s neurological status. If this is the case, the investigator will be notified and after evaluation of the CT scan and the neurological examination and NIHSS, a primary outcome point will be adjudicated. We will use the symptomatic intracerebral hemorrhage definition from both ECASS 3 as well as the original NINDS IV rt-PA trial criteria for comparison.

- In ECASS 3 symptomatic intracerebral hemorrhage is defined as: Any extravascular blood in the brain or within the cranium associated with a clinical deterioration defined as an increase in 4 points on the NIHSS, or that lead to death and was identified as the predominant cause of neurological deterioration.
- In the original NINDS IV rt-PA trial symptomatic intracerebral hemorrhage is defined as: A hemorrhage not seen in previous CT scans and there was a suspicion of hemorrhage or any deterioration in neurologic status.

The subject will receive standard care for stroke and will be discharged from the hospital, when medically stable, to the appropriate rehabilitation setting. All patients will return for a follow up visit at 90 days and will receive a modified Rankin score, NIHSS and Barthel index.

The subject’s involvement with this study will end at 90 days after the assessment.

- 1. **Rationale for Study**

Fink et al (Stroke 2002) and Nadeau et al (Can J Neurol Sci 2005) found that 13% to 27% of patients with acute stroke wake up with symptoms.

Patients that wake up with symptoms of acute stroke are currently not treated with IV rt-PA. This is a group that has not previously been the focus of clinical trials testing acute stroke therapy. **Our hypothesis** is that a significant number of patients that wake up with stroke symptoms may have developed the stroke at the time of awakening, and may be within the 4.5 hour window if they arrive to the Emergency Department within that time, therefore IV tPA should be safe and effective in this population.

Based on our hypothesis, which presumes that patients presenting within four and half hours of waking up with stroke symptoms, actually had their stroke onset within that time window, treatment with tPA will follow the standard guidelines for treatment within 4.5 hours of known onset or time from last see well. This protocol is described above in section 3.1.

- 1. Outcome Measures

3.3.1 Primary Outcome Measure

This is a safety study; therefore the primary outcome is a safety outcome:

- Symptomatic intracerebral hemorrhage rate within 36 hours of treatment. By ECASS 3 and NINDS rt-PA study as stated above.

3.3.2 Secondary Outcome Measures

Secondary outcome measures will be to explore efficacy:

- Modified Rankin score, NIHSS, Barthel index at 90 days.
- NIHSS at 24hrs.

3.3.3 Safety Outcome Measures

The following safety outcome measures will be evaluated:

- Asymptomatic intracerebral hemorrhage occurring at any time during the study.
- Symptomatic intracarebral hemorrhage occurring at any time during the study.
- Mortality at 90 days.

3.4 Safety Plan

The adverse events associated with systemic use of Activase are well described and consist primarily of bleeding complications, including serious major hemorrhagic events and ICH. The incidence of these bleeding complications has been quantified in patients receiving relatively large doses Activase for acute myocardial infarction, acute ischemic stroke, and pulmonary embolism. Any potential bleeding attributable to Activase is most likely to occur within 24 hours of treatment and is unlikely to occur after 72 hours.

See Section 5 (Assessment of Safety) for complete details of the safety evaluation for this study.

3.5 Compliance with Laws and Regulations

This study will be conducted in accordance with current U.S. Food and Drug Administration (FDA) Good Clinical Practices (GCPs), and local ethical and legal requirements.

4.0 MATERIALS AND METHODS

- 1. Subjects

4.1.1 Subject Selection

All sites for this study will be certified stroke centers (The Johns Hopkins Hospital and Johns Hopkins Bayview Medical Center). Stroke center personnel will be made aware of this trial and will be instructed to identify patients with wake up strokes and to call the acute stroke team. Patients presenting to the ED will be identified upon arrival as acute stroke patients and screened by the ED personnel for time of onset (this is standard procedure). The stroke team will evaluate the patients with a history and neurological exam as well as EKG, laboratory studies and a non-contrast head CT or MRI per the standard of care at the clinical site. Starting on April 29, 2012, the standard brain imaging evaluation for all acute stroke patients without a defined time of less than 4.5 hors of last seen well will be an MRI of the brain. Patients waking up with stroke symptoms and presenting within four and half hours of awakening will be identified and the investigator will be notified. The patient will be screened for the presence of inclusion criteria and absence of exclusion criteria, and if found to qualify for the study will be asked to participate after reviewing the details of the study and consent form.

There will be a screening log that will include information about the patients screened and the reason for non-enrollment. Patients that decline enrollment for treatment will be approached for participation in a de-identified database to capture their outcomes. This group will serve as a comparison group.

The investigator will explain the clinical trial to the patient or the Legally Authorized Representative (LAR), and will review the consent form, answering any questions. This will be conducted in a private and calm setting as much as it is possible in the ED. The patient or LAR will be given the consent form to review and a copy of signed consent form as well. The subjects will receive treatment within 4.5hrs from waking up with stroke symptoms.

Subjects will receive a subject ID that will be a sequential numerical code.

4.1.2 Inclusion Criteria

Subjects will be eligible if the following criteria are met:

- Ability to provide written informed consent (or a LAR available to provide informed consent) and comply with study assessments for the full duration of the study.
- Age > 18 years

Patient‑related considerations

1. Patients to be included will be diagnosed as having an acute ischemic stroke by history and physical exam.
2. The subjects of this study should have woken up with symptoms of stroke and present to the Emergency Department (ED) within 4.5 hours of awakening.
3. NIHSS > 3
4. A non-contrast head CT without hemorrhage and without hypodensity more than 1/3 of the MCA territory; or MRI demonstrating no hemorrhage, and with a DWI lesion no greater than 70mL and FLAIR without a well demarcated hyperintense lesion that is more than 1/3 of the MCA territory.
5. Pre morbid modified Rankin score of 0 or 1.
6. Treatment with IV rt-PA to be initiated prior to 4.5 hours of waking up with acute stroke symptoms.

Other considerations

1. Some patients will not be able to provide informed consent. In these cases consent will be obtained from the Legally Authorized Representative (LAR).
2. Patients that in the judgment of the investigator will not be able to comply with the follow up examinations due to known non-compliance, residence is distant, terminal illness, etc; will not be candidates for inclusion in the trial.

4.1.3 Exclusion Criteria

The exclusion criteria include current exclusion criteria for the use of rt-PA in acute stroke within 3 hours of onset and are listed below:

a. Rapidly improving deficit to an NIHSS < =3.

b. Sustained SBP>185, DBP>110 despite treatment.

c. Glucose < 50 mg/dL.

d. Stroke or head trauma within the last 3 months.

e. History of intracranial hemorrhage. Symptoms of subarachnoid hemorrhage.

f. Major surgery within 14 days.

g. GI/GU hemorrhage within 21 days.

h. INR > 1.7.

i. Heparin within 48 hours with an elevated PTT.

j. Platelet count < 100.000.

k. Presumed septic embolus, or suspicion of bacterial endocarditis.

l. Presumed pericarditis including pericarditis after myocardial infarction.

m. Suspicion of aortic dissection.

n. Use of anticoagulants such as dabigatran, rivaroxaban, apixaban, enoxaparin.

Additional exclusion criteria:

- Pregnant or lactating women will not be eligible. This is due to the risks involved for the fetus or lactating child, rt-PA is class C for pregnancy and its risk in lactation are unknown. Regarding becoming pregnant, there will be no specific contraindication for this as it is something that would happen after the treatment phase of the study. All women on childbearing age will be tested for pregnancy prior to enrollment.
- Patients taking low molecular heparin, direct thrombin inhibitors, or factor Xa inhibitors will be excluded from the study due to the risk of bleeding.
- Patients with a known allergy or sensitivity to rt-PA will be excluded.
- Patients with a pre morbid modified Rankin score higher than 2 will be excluded as it will make it difficult to assess for outcomes and it will be difficult to assure compliance with follow up. Children (younger than 18 years of age) will be excluded from the study, as there are no confirmatory trials of rt-PA in acute stroke in this population. Patients with a pre existing disease that limits their life expectancy to less than 6 months will be excluded as well.
- Inability or unwillingness of subject or legal guardian/representative to give written informed consent.
- Any condition that to the judgment of the investigator will increase risk unnecessarily.
  1. Method of Treatment Assignment

All patients enrolled will receive IV tPA. This is an open label, single arm study.

- 1. Study Treatment

Activase will be administered at the standard dose for treatment of acute stroke: 0.9mg/kg, 10% IV bolus, and the rest by IV continuous infusion over one hour. Maximum dose is 90mg.

- - 1. Formulation (Alteplase and Other Drugs)

Activase is a sterile, white to pale yellow, preservative-free lyophilized powder for intravenous administration after reconstitution with sterile water for injection, USP. Activase is supplied in 50-mg vials containing vacuum and in 100mg vials without vacuum. It is important that Activase be reconstituted only with Sterile Water For Injection, USP, without preservatives. Do not use Bacteriostatic Water for Injection, USP. The reconstituted preparation results in a colorless to pale yellow transparent solution containing Activase 1mg/ml at approximately pH 7.3.

For further details, see the Activase Package Insert.

- - 1. Dosage, Administration and Storage

a. Dosage

The dose of Activase will be administered as outlined (See Treatment Plan 4.3). If serious bleeding occurs the infusion of Activase should be terminated immediately. In the event of severe or life-threatening anaphylaxis or hypersensitivity reaction, discontinue the patient from the treatment phase. Do not retreat with Activase.

See the WARNINGS section of the Package Insert for language for management of adverse reactions.

b. Administration

See Activase Package Insert for detailed prescribing information.

c. Storage

Store lyophilized Activase at controlled room temperature not to exceed 30°C (86°F) or under refrigeration 2–8°C (36–46°F). Protect the lyophilized material during extended storage from excessive exposure to light.

Do not use beyond the expiration date stamped on the vial.

- - 1. **Dose Modifications**

There will be no dose modification except for discontinuing treatment if there is an adverse event, i.e., bleeding complication or anaphylactic reaction.

- 1. Concomitant Therapy AND EXCLUDED THERAPY

No antithrombotic therapy including aspirin, warfarin, heparin, enoxaparin, Clopidogrel, are allowed in the first 24 hours after treatment with Activase. After 24 hours, the treating physician will choose antithrombotic therapy.

Subjects may continue to receive all medications and standard treatments administered for their conditions at the discretion of their treating physician.

4.5 Study Assessments

Interventions, Administration, and Duration

Once the patient has agreed to participate and has signed the consent form, a subject ID number will be assigned. Prior to be enrolled in the study, all subjects will have received the following procedures/tests:

1. History and physical examination.
2. EKG, CBC, electrolytes, creatinine and BUN, PT and PTT.
3. Two intravenous catheters will be started.
4. A non-contrast CT scan of the brain or MRI as per the standard of care at the clinical site.

The subject will then receive open label rt-PA via IV catheter at a dose of 0.9mg/kg, 10% given as a bolus and the remainder given as a continuous infusion over one hour. The maximum dose is 90mg.

The study subject will be monitored in the ED until the completion of the rt-PA infusion, following the standard protocol for monitoring after rt-PA administration for acute stroke within 4.5 hours of onset. Once the infusion is completed, the subject will be transferred to the intensive care unit where the observation as per the standard care protocol will be followed. Blood pressure and neurological status will be closely monitored as per protocol. Observation in the intensive care unit will be for a minimum of 24 hours. Subjects will be under the care of the admitting physician that may also be the study investigator.

A non-contrast head CT will be done between 20-30 hours after administration of rt-PA and at any time before or after if there is the clinical suspicion of hemorrhagic transformation or a decline in the subject’s neurological status. If this is the case, the investigator will be notified and after evaluation of the CT scan and the neurological examination and NIHSS, a primary outcome point will be adjudicated.

The subject will receive standard care for stroke and will be discharged from the hospital, when medically stable, to the appropriate rehabilitation setting. All patients will return for a follow up visit at 90 days and will receive a modified Rankin score, NIHSS and Barthel index.

The subject’s involvement with this study will end at 90 days after the assessment.

Schedule of Evaluations

| Evaluation | Screening  (Day 1) | Entry  (Day1) | 24 hrs | 36  hrs | 90 days |
| --- | --- | --- | --- | --- | --- |
| Informed Consent |  | X |  |  |  |
| Documentation of Disease/Disorder | X |  |  |  |  |
| Medical/Treatment History | X |  |  |  |  |
| Adverse Event Ascertainment |  | X | X | X | X |
| Clinical Assessment | X | X | X | X | X |
| Hematology | X |  |  |  |  |
| Chemistry | X |  |  |  |  |
| Non-Contrast Head CT or MRI | X | X | X |  |  |
| Pregnancy Testing | X |  |  |  |  |
| Questionnaires |  |  |  |  | X |

4.5.1 Screening and Pre-treatment Assessments

These evaluations occur prior to the subject receiving any study interventions.

Screening

Patients will be screened for the study upon arrival to the ED. The stroke team should be alerted and arrive at the bedside within 15 minutes of the patient’s arrival and the investigator will be alerted. If the patient is a candidate for the study, the investigator will invite the patient to participate in the study as soon as possible keeping in mind the need to give the study medication within four and half hours of waking up with symptoms. The patient will receive the standard ED evaluation for acute stroke including:

1. History and physical examination.
2. EKG, CBC, electrolytes, creatinine and BUN, PT and PTT.
3. Two intravenous catheters will be started.
4. A non-contrast CT scan of the brain or MRI per standard of care at the clinical site.

Entry/Baseline

The subject will be consented within 4.5 hours of waking up with stroke. After consent is signed, study drug will be administered.

Informed Consent

Patient or LAR will be approached in a calm and private setting, as much as possible. The investigator will describe the study. The consent form will be explained, time allowed for reading of the form and a copy will be given after signing. A copy of the consent form will remain in the subject’s medical record and in the study archives.

- - 1. Assessments During Treatment

Standard protocol for IV rtPA administration for acute stroke within 4.5 hours will be followed. This includes assessment of neurological status and vital signs every 15 minutes for the first two hours, every 30 minutes for the next two hours and ever hour for the next 16 hours.

Subjects will have a non-contrast head CT between 20-30 hours post rt-PA administration and at any time that there is a clinical deterioration. At 24 hours an NIHSS will be performed and the subject assessed for adverse events and serious adverse events. An NIHSS will be performed if there is a clinical deterioration in addition to the CT scan. Subjects will return for an evaluation 90 days after administration of study drug and will have an NIHSS, Barthel index and modified Rankin score. The occurrence of adverse events and serious adverse events will be assessed during all contacts with the subjects.

4.5.3 Follow-Up Assessments

The NIHSS will be used to measure stroke severity at the screening point, at 24 hours after treatment, in the event of clinical deterioration and at 90 days after study drug administration. The Barthel index and modified Rankin score will be administered at 90 days.

- 1. Subject Discontinuation

Subjects have a right to withdraw from the study at any time. Subjects that decide to withdraw from the study within the first 24 hours, will need to have their CT scan at 20-30 hours, and continue observation in the intensive care unit, will not receive antithrombotic therapy during the first 24 hours as per standard of care. No data will be collected on the patient after voluntary withdrawal from the study.

The subject may be withdrawn from the study for any reasons: if it is in the best interest of the subject, intercurrent illness, adverse events, or worsening condition. The IRB, NINDS, Genentech, the OHRP, the FDA, or other government agencies as part of their duties to ensure that research subjects are protected, may request the withdrawal of a subject because of protocol violations, administrative reasons, or any other valid and ethical reasons.

- 1. **Study Discontinuation**

The IRB, NINDS, Genentech, the OHRP, the FDA, or other government agencies as part of their duties to ensure that research subjects are protected, at any time may terminate this study. Reasons for terminating the study may include the following:

- The incidence or severity of adverse events in this or other studies indicates a potential health hazard to subjects
- Subject enrollment is unsatisfactory
- Data recording is inaccurate or incomplete
  1. **STATISTICAL METHODS**

4.8.1 Analysis of the Conduct of the Study

The study proposed has a single arm, open label study to test the safety of IV tPA in stroke upon awakening. The null hypothesis (Ho) is that IV rt-PA in this population will have a symptomatic intracerebral hemorrhage rate of less than 6%.

The alternative hypothesis (Ha) is that IV rt-PA will have a symptomatic intracerebral hemorrhage rate of more than 10%, which would be unacceptably high.

This is a proof of concept study. We will obtain the frequency of the primary outcome (symptomatic intracerebral hemorrhage within the first 36 hours).

- - 1. Baseline Demographics

The sample size will be 20 patients, to assess the safety of rt-PA in this population. The number of patients with acute stroke treated at Johns Hopkins Hospital (JHH) and Johns Hopkins Bayview Medical Center (JHBMC) from February 1, 2008 to January 31, 2009 was 175 and 196 respectively. Assuming that 12% may present with stroke symptoms upon awakening and be eligible by CT criteria (Based on a retrospective evaluation of one year of stroke patients at JHH), with a rate of agreement to participate of 60%; this would yield 12 and 14 study subjects per year at JHH and JHBMC respectively. Based on this assessment, this study can be completed in one year.

- - 1. Safety Analysis

Because this study is designed to assess safety, the safety analysis is the same as the primary outcome analysis, which is the assessment of the rate of symptomatic intracerebral hemorrhage after IV rt-PA in patients that present to the ED within 4.5 hours of waking up with stroke. A DSMB (composed of one member independent of the investigator team) will conduct an analysis of the rate of symptomatic intracerebral hemorrhage by ECASS 3 criteria.

Symptomatic intracerebral hemorrhages (events) will be evaluated after each subject completes therapy. During the phase of the study in which there are 1-10 subjects enrolled, the study will stop at any point there are two events. During the phase of the study in which there are 11-20 subjects enrolled, the study will stop at any point if there are three events.

4.8.4 Efficacy Analysis

a. Primary Endpoint

Symptomatic intracerebral hemorrhage within 36 hours of treatment is the main safety outcome measure in clinical trials of thrombolytic therapy in acute stroke and is the primary outcome measure for this trial. (The ATLANTIS, ECASS, and NINDS rt-PA Study Group Investigators (Lancet 2004), Hacke W, et al (NEJM 2008).)

We will use the symptomatic intracerebral hemorrhage definition from both ECASS 3 as well as the original NINDS IV rt-PA trial criteria for comparison.

- In ECASS 3 symptomatic intracerebral hemorrhage is defined as: Any extravascular blood in the brain or within the cranium associated with a clinical deterioration defined as an increase in 4 points on the NIHSS, or that lead to death and was identified as the predominant cause of neurological deterioration.
- In the original NINDS IV rt-PA trial symptomatic intracerebral hemorrhage is defined as: A hemorrhage not seen in previous CT scans and there was a suspicion of hemorrhage or any deterioration in neurologic status.

b. Secondary Endpoints

1. Asymptomatic intracerebral hemorrhage at any time during the study.

2. Symptomatic intracerebral hemorrhage at any time during the study.

3. Efficacy measures:

a. Modified Rankin score, NIHSS, Barthel index at 90 days

b. NIHSS at 24hrs

c. Mortality at 90 days.

4.9 Data Quality Assurance

All data collected will be kept in password-protected computers. Paper documents will be in secure locations. All data will be kept under the subject’s ID and will not be referenced to the subject’s identifiers. We will not destroy study data.

Data management at the clinical site and coordinating center will be conducted according to Good Clinical Practices.

Clinical sites will be monitored every 6 months to assure quality of the data, compliance with regulations and to verify source documents.

5. ASSESSMENT OF SAFETY

5.1 Specification of Safety Variables

Safety assessments will consist of monitoring and reporting adverse events (AEs) and serious adverse events (SAEs) that are considered related to **ACTIVASE**, all events of death, and any study specific issue of concern.

5.1.1 Adverse Events

An AE is any unfavorable and unintended sign, symptom, or disease temporally associated with the use of an investigational (medicinal) product or other protocol-imposed intervention, regardless of attribution.

This includes the following:

- AEs not previously observed in the subject that emerge during the protocol-specified AE reporting period, including signs or symptoms associated with ischemic stroke that were not present prior to the AE reporting period.
- Complications that occur as a result of protocol-mandated interventions (e.g., invasive procedures such as cardiac catheterizations).
- If applicable, AEs that occur prior to assignment of study treatment associated with medication washout, no treatment run-in, or other protocol-mandated intervention.
- Preexisting medical conditions (other than the condition being studied) judged by the investigator to have worsened in severity or frequency or changed in character during the protocol-specified AE reporting period.

5.1.2 Serious Adverse Events

An AE should be classified as an SAE if:

It results in death (i.e., the AE actually causes or leads to death).

It is life threatening (i.e., the AE, in the view of the investigator, places the subject at immediate risk of death. It does not include an AE that, had it occurred in a more severe form, might have caused death.).

It requires or prolongs inpatient hospitalization.

It results in persistent or significant disability/incapacity (i.e., the AE results in substantial disruption of the subject’s ability to conduct normal life functions).

It results in a congenital anomaly/birth defect in a neonate/infant born to a mother exposed to the investigational product.

It is considered a significant medical event by the investigator based on medical judgment (e.g., may jeopardize the subject or may require medical/surgical intervention to prevent one of the outcomes listed above).

5.2 Methods and Timing for Assessing AND Recording Safety variables

The investigator is responsible for ensuring that all AEs and SAEs that are observed or reported during the study, as outlined in Section 5.1.1, are collected and reported to the FDA, appropriate IRB(s), and Genentech, Inc. in accordance with CFR 312.32 (IND Safety Reports).

5.2.1 Adverse Event Reporting Period

The study period during which all AEs and SAEs must be reported begins after informed consent is obtained and initiation of study treatment and ends 90 days following the last administration of study treatment or study discontinuation/termination, whichever is earlier. After this period, investigators should only report SAEs that are attributed to prior study treatment.

5.2.2 Assessment of Adverse Events

All AEs and SAEs whether volunteered by the subject, discovered by study personnel during questioning, or detected through physical examination, laboratory test, or other means will be reported appropriately.

Each reported AE or SAE will be described by its duration (i.e., start and end dates), regulatory seriousness criteria if applicable, suspected relationship to **ALTEPLASE** (see following guidance), and actions taken.

To ensure consistency of AE and SAE causality assessments, investigators should apply the following general guideline:

Yes

There is a plausible temporal relationship between the onset of the AE and administration of **ALTEPLASE**, and the AE cannot be readily explained by the subject’s clinical state, intercurrent illness, or concomitant therapies; and/or the AE follows a known pattern of response to **ALTEPLASE**; and/or the AE abates or resolves upon discontinuation of **ALTEPLASE** or dose reduction and, if applicable, reappears upon re-challenge.

No

Evidence exists that the AE has an etiology other than **ALTEPLASE** (e.g., preexisting medical condition, underlying disease, intercurrent illness, or concomitant medication); and/or the AE has no plausible temporal relationship to **ALTEPLASE** administration (e.g., cancer diagnosed 2 days after first dose of study drug).

Expected adverse events are those adverse events that are listed or characterized in the Package Insert or current Investigator Brochure.

Unexpected adverse events are those not listed in the Package Insert (P.I.) or current Investigator Brochure (I.B.) or not identified. This includes adverse events for which the specificity or severity is not consistent with the description in the P.I. or I.B. For example, under this definition, hepatic necrosis would be unexpected if the P.I. or I.B. only referred to elevated hepatic enzymes or hepatitis.

5.3 Procedures for Eliciting, Recording, and Reporting Adverse Events

5.3.1 Eliciting Adverse Events

All patients enrolled will have a non-contrast head CT within 20-30 hours post treatment with ACTIVASE or if there is any change or deterioration in neurological status. Presence of hemorrhagic transformation in the CT will be classified as symptomatic if there is a concomitant deterioration in the patient’s neurologic status and as asymptomatic if there isn’t any change.

During the 24-hour, 36 hour and 90 days visit, adverse events will be elicited by asking for new symptoms, diagnosis, changes in health, since the last visit.

5.3.2 Specific Instructions for Recording Adverse Events

Investigators should use correct medical terminology/concepts when reporting AEs or SAEs. Avoid colloquialisms and abbreviations.

a. Diagnosis vs. Signs and Symptoms

If known at the time of reporting, a diagnosis should be reported rather than individual signs and symptoms (e.g., record only liver failure or hepatitis rather than jaundice, asterixis, and elevated transaminases). However, if a constellation of signs and/or symptoms cannot be medically characterized as a single diagnosis or syndrome at the time of reporting, it is ok to report the information that is currently available. If a diagnosis is subsequently established, it should be reported as follow-up information.

b. Deaths

All deaths that occur during the protocol-specified AE reporting period (see Section 5.1.2), regardless of attribution, will be reported to the appropriate parties. When recording a death, the event or condition that caused or contributed to the fatal outcome should be reported as the single medical concept. If the cause of death is unknown and cannot be ascertained at the time of reporting, report “Unexplained Death”.

c. Preexisting Medical Conditions

A preexisting medical condition is one that is present at the start of the study. Such conditions should be reported as medical and surgical history. A preexisting medical condition should be re-assessed throughout the trial and reported as an AE or SAE only if the frequency, severity, or character of the condition worsens during the study. When reporting such events, it is important to convey the concept that the preexisting condition has changed by including applicable descriptors (e.g., “more frequent headaches”).

d. Hospitalizations for Medical or Surgical Procedures

Any AE that results in hospitalization or prolonged hospitalization should be documented and reported as an SAE. If a subject is hospitalized to undergo a medical or surgical procedure as a result of an AE, the event responsible for the procedure, not the procedure itself, should be reported as the SAE. For example, if a subject is hospitalized to undergo coronary bypass surgery, record the heart condition that necessitated the bypass as the SAE.

- Hospitalizations for the following reasons do not require reporting:
- Hospitalization or prolonged hospitalization for diagnostic or elective surgical procedures for preexisting conditions
- Hospitalization or prolonged hospitalization required to allow efficacy measurement for the study or
- Hospitalization or prolonged hospitalization for scheduled therapy of the target disease of the study.

e. Pregnancy

Pregnancy test is required prior to enrolment in the study. Pregnancy is an exclusion criterion for this study. Because the intervention is brief (one hour administration/infusion), no female subject will become pregnant while receiving the investigational therapy. If a female subject becomes pregnant within 90 days after the last dose of study drug, a report should be completed and expeditiously submitted to the Genentech, Inc. Follow-up to obtain the outcome of the pregnancy should also occur. Abortion, whether accidental, therapeutic, or spontaneous, should always be classified as serious, and expeditiously reported as an SAE. Similarly, any congenital anomaly/birth defect in a child born to a female subject exposed to **ALTEPLASE** should be reported as an SAE.

f. Post-Study Adverse Events

The investigator should expeditiously report any SAE occurring after a subject has completed or discontinued study participation if attributed to prior **ALTEPLASE** exposure. If the investigator should become aware of the development of cancer or a congenital anomaly in a subsequently conceived offspring of a female subject who participated in the study, this should be reported as an SAE.

g. Reconciliation

The Sponsor agrees to conduct reconciliation for the product. Genentech and the Sponsor will agree to the reconciliation periodicity and format, but agree at minimum to exchange monthly line listings of cases received by the other party. If discrepancies are identified, the Sponsor and Genentech will cooperate in resolving the discrepancies. The responsible individuals for each party shall handle the matter on a case-by-case basis until satisfactory resolution.

h. SAE Reporting

Investigators must report all SAEs to Genentech within the timelines described below.

The completed Medwatch/case report should be faxed immediately upon completion to Genentech Drug Safety at:

(650) 225‑4682
or
(650) 225‑5288

- Relevant follow-up information should be submitted to Genentech Drug Safety as soon as it becomes available.
- Serious AE reports that are related to ALTEPLASE will be transmitted to Genentech within fifteen (15) calendar days of the Awareness Date.
- Serious AE reports that are unrelated to ALTEPLASE will be transmitted to Genentech within thirty (30) calendar days of the Awareness Date.
- Additional Reporting Requirements to Genentech include the following:
- Any reports of pregnancy following the start of administration with the ALTEPLASE will be transmitted to Genentech within thirty (30) calendar days of the Awareness Date.
- All Non-serious Adverse Events originating from the Study will be forwarded, at most, on a quarterly report to Genentech.

Note: Investigators should also report events to their IRB as required.

### MedWatch 3500A Reporting Guidelines

In addition to completing appropriate patient demographic and suspect medication information, the report should include the following information within the Event Description (section 5) of the MedWatch 3500A form:

- - Protocol description (and number, if assigned)
  - Description of event, severity, treatment, and outcome if known
  - Supportive laboratory results and diagnostics
  - Investigator’s assessment of the relationship of the adverse event to each investigational product and suspect medication

#### Follow-up Information

Additional information may be added to a previously submitted report by any of the following methods:

- - Adding to the original MedWatch 3500A report and submitting it as follow-up
  - Adding supplemental summary information and submitting it as follow-up with the original MedWatch 3500A form
  - Summarizing new information and faxing it with a cover letter including patient identifiers (i.e. D.O.B. initial, patient number), protocol description and number, if assigned, brief adverse event description, and notation that additional or follow-up information is being submitted (The patient identifiers are important so that the new information is added to the correct initial report)

Occasionally Genentech may contact the reporter for additional information, clarification, or current status of the patient for whom and adverse event was reported. For questions regarding SAE reporting, you may contact the Genentech Drug Safety representative noted above or the MSL assigned to the study. Relevant follow-up information should be submitted to Genentech Drug Safety as soon as it becomes available and/or upon request.

MedWatch 3500A (Mandatory Reporting) form is available at
<http://www.fda.gov/Safety/MedWatch/HowToReport/DownloadForms/default.htm>

5.4 Additional Reporting Requirements for IND Holders

For Investigator-Sponspored IND Studies, some additional reporting requirements for the FDA apply in accordance with the guidance set forth in 21 CFR § 600.80.

Events meeting the following criteria need to be submitted to the Food and Drug Administration (FDA) as expedited IND Safety Reports according to the following guidance and timelines:

**7 Calendar Day Telephone or Fax Report:**

The Investigator is required to notify the FDA of any **fatal or life-threatening** adverse event that is **unexpected** and assessed by the investigator to be **possibly related** to the use of **ALTEPLASE**. An unexpected adverse event is one that is not already described in the **ALTEPLASE** US PI. Such reports are to be telephoned or faxed to the FDA and Genentech within 7 calendar days of first learning of the event.

**15 Calendar Day Written Report**

The Investigator is also required to notify the FDA and all participating investigators, in a written IND Safety Report, of any **serious, unexpected** AE that is considered reasonably or **possibly related** to the use of **ALTEPLASE**. An **unexpected** adverse event is one that is not already described in the **ALTEPLASE** US PI.

• Written IND Safety reports should include an **Analysis of Similar Events** (see Appendix D for template) in accordance with regulation 21 CFR § 312.32. All safety reports previously filed by the investigator with the IND concerning similar events should be analyzed and the significance of the new report in light of the previous, similar reports commented on.

• Written IND safety reports with Analysis of Similar Events are to be submitted to the FDA, Genentech, and all participating investigators within 15 calendar days of first learning of the event. The FDA prefers these reports on a Medwatch 3500 form, but alternative formats are acceptable (e.g., summary letter).

**FDA fax number for IND Safety Reports:**

1 (800) FDA 0178

**All written IND Safety Reports submitted to the FDA by the Investigator must also be faxed to:**

Genentech Drug Safety at:

(650) 225‑4682
or
(650) 225‑5288

***AND***

Site IRB: Johns Hopkins

Phone: 410-955-3008

Fax: 410-955-4367

**For questions related to safety reporting, please contact:**

Genentech Drug Safety

Tel: (888) 835-2555

Fax: (650) 225-4682 or (650) 225-5288

**Copies to Genentech:**

All IND annual reports submitted to the FDA by the Sponsor-Investigator should be copied to Genentech. Copies of such reports should be faxed to:

Genentech Drug Safety

Tel: (888) 835-2555

Fax: (650) 225-4682 or (650) 225-5288

5.5 Study Close-Out

**Any study report submitted to the FDA by the Sponsor-Investigator should be copied to Genentech.** This includes all IND annual reports and the Clinical Study Report (final study report). Additionally, any literature articles that are a result of the study should be sent to Genentech.

Copies of such reports should be faxed to the assigned Clinical Operations Contact for the study:

Lytics IST Program Fax: 866.283.2263

6.0 INVESTIGATOR REQUIREMENTS

6.1 Study Initiation

Before the start of this study, the following documents must be on file with Johns Hopkins Medicine or its appointed representative:

- FDA correspondence letter assigning an IND number or an IND waiver letter
- Original U.S. FDA Form 1572 (for all studies conducted under U.S. Investigational New Drug [IND] regulations), signed by the Principal Investigator

The names of any sub-investigators must appear on this form. Investigators must also complete all regulatory documentation as required by local and national regulations.

- Current curricula vitae of the Principal Investigator
- Medical License
- Written documentation of IRB approval of protocol (identified by Johns Hopkins Medicine *IRB,* protocol number or title and date of approval) and informed consent document (identified by Johns Hopkins Medicine IRB, protocol number or title and date of approval)
- A copy of the IRB‑approved informed consent document
- A copy of the IRB approved Protocol
- Documentation of registration into clinical research website (e.g., [www.clinicaltrials.gov](http://www.clinicaltrials.gov/)) (as applicable)
- Fully executed contract
- Investigator Brochure signature receipt
- Written documentation of IRB review and approval of any advertising materials to be used for study recruitment, if applicable

The informed consent document and any advertising materials must also be reviewed and approved by the Johns Hopkins Medicine Legal Department.

- Certified translations of IRB approval letters, pertinent correspondence, and approved informed consent document (when applicable)
- Current laboratory certification of the laboratory performing the analysis as well as current normal laboratory ranges for all laboratory tests.

6.2 Study Completion

The following data and materials are required by Genentech, Inc before a study can be considered complete or terminated:

- Laboratory findings, clinical data, and all special test results from screening through the end of the study follow‑up period (if applicable)
- Case Report Forms properly completed by appropriate study personnel and signed and dated by the investigator (if applicable)
- Copies of protocol amendments and IRB approval/notification (if applicable)
- A summary of the study prepared by the Principal Investigator (will accept IRB summary close letter) (if applicable)
- All regulatory documents (e.g., curricula vitae for each Principal Investigator, U.S. FDA Form 1572)
  1. Informed Consent

Informed consent documents will be provided to each subject.

The informed consent document must be signed and dated by the subject or the subject’s legally authorized representative before his or her participation in the study. The case history for each subject shall document that informed consent was obtained prior to participation in the study. A copy of the informed consent document must be provided to the subject or the subject's legally authorized representative. If applicable, it will be provided in a certified translation of the local language.

Signed consent forms must remain in each subject’s study file and must be available for verification at any time.

The following basic elements must be included:

- A statement that the study involves research, an explanation of the purposes of the research and the expected duration of the patient’s participation, a description of the procedures to be followed, and identification of any procedures or drug used for purposes which are experimental
- A description of any reasonably foreseeable risks or discomforts to the patients
- A description of any benefits to the patient or to others, which may reasonably be expected from the research. A description that there may be no benefit from this research.
- A disclosure of appropriate alternative procedures or courses of treatment, if any, that might be advantageous to the patient
- A statement describing the extent, if any, to which confidentiality records identifying the patient will be maintained and that notes the possibility that the FDA and Johns Hopkins Medicine and the drug manufacturer may inspect the records
- For research involving more than minimal risk, an explanation as to whether any compensation and any medical treatments are available should injury occur and, if so, what they consist of or where further information may be obtained
- An explanation of whom to contact for answers to pertinent questions about the research and research patient’s rights, and whom to contact in the event of a research-related injury to the patient
- A statement that participation is voluntary, that refusal to participate will involve no penalty or loss of benefits to which the patient is otherwise entitled, and that the patient may discontinue participation at any time without penalty or loss of benefits to which the patient is otherwise entitled

6.4 Institutional Review Board or Ethics Committee Approval

This protocol, the informed consent document, and relevant supporting information must be submitted to the IRB/EC for review and must be approved before the study is initiated. The study will be conducted in accordance with U.S. FDA, applicable national and local health authorities, and IRB/EC requirements.

The Principal Investigator is responsible for keeping the IRB/EC apprised of the progress of the study and of any changes made to the protocol as deemed appropriate, but in any case the IRB/EC must be updated at least once a year. The Principal Investigator must also keep the IRB/EC informed of any significant adverse events.

Investigators are required to promptly notify their respective IRB/EC of all adverse drug reactions that are both serious and unexpected. This generally refers to serious adverse events that are not already identified in the Investigator Brochure and that are considered possibly or probably related to the study drug by the investigator. Some IRBs or ECs may have other specific adverse event requirements that investigators are expected to adhere to. Investigators must immediately forward to their IRB/EC any written safety report or update provided by Genentech (e.g., IND safety report, Investigator Brochure, safety amendments and updates, etc.).

6.5 Case Report Forms

All CRFs should be filled out completely by appropriate personnel. The CRF should be reviewed, signed, and dated by the investigator.

All CRFs should be completed in a neat, legible manner to ensure accurate interpretation of data. Black ink is required to ensure clarity of reproduced CRF copies. When making changes or corrections, cross out the original entry with a single line, and initial and date the change. DO NOT ERASE, OVERWRITE, OR USE CORRECTION FLUID ON THE ORIGINAL.

6.6 Study Drug Accountability

The Investigator is responsible for the control and distribution of study drug.

All partially used or empty containers should be disposed of at the study site according to institutional standard operating procedure.

6.7 Disclosure of Data

Subject medical information obtained by this study is confidential, and disclosure to third parties other than those noted below is prohibited.

Upon the subject’s permission, medical information may be given to his or her personal physician or other appropriate medical personnel responsible for his or her welfare.

Data generated by this study must be available for inspection upon request by representatives of the U.S. FDA, national and local health authorities, the drug manufacturer and the IRB/EC for each study site, if appropriate.

6.8 Retention of Records

U.S. FDA regulations (21 CFR §312.62[c]) require that records and documents pertaining to the conduct of this study and the distribution of investigational drug, including CRFs, consent forms, laboratory test results, and medication inventory records, must be retained by the Principal Investigator for 2 years after the investigation is discontinued and the U.S. FDA and the applicable national and local health authorities are notified.

**REFERENCES**

1. The National Institute Of Neurological Disorders And Stroke rt-PA Stroke Study Group. Tissue Plasminogen Activator for Acute Ischemic Stroke. NEJM. 1995;333(4):1581-1587.

2. Fink JN, Kumar S, Horkan C, Linfante I, Selim MH, Caplan LR, Schlaug G. The stroke patient who woke up: clinical and radiological features, including diffusion and perfusion MRI. *Stroke.* 2002;33**:** 988-93.

3. Nadeau JO, Fang J, Kapral MK, Silver FL, Hill MD (2005). Outcome after stroke upon awakening. *Can J Neurol Sci.* 2005;32: 232-6.

4. Todo K, Moriwaki H, Saito K, Tanaka M, Oe H, Naritomi H. Early CT findings in unknown-onset and wake-up strokes. *Cerebrovasc Dis.* 2006;21: 367-71.

5. Barreto AD, Martin-Schild S, Hallevi H, Morales MM, Abraham AT, Gonzales NR, Illoh K, Grotta JC, Savitz SI. Thrombolytic Therapy for Patients Who Wake-Up With Stroke. *Stroke.* 2009;40:827-832.

6. Hacke W, Kaste M, Bluhmki E, Brozman M, Davalos A, Guidetti D, Larrue V, Lees KR, Medghri Z, Machnig T, Schneider D, von Kummer R, Wahlgren N, Toni D. Thrombolysis with Alteplase 3 to 4.5 Hours after Acute Ischemic Stroke. *N Engl J Med.* 2008;359(13):1317-1329.

7. Deamaerschalk BM, Yip TR. Economic Benefit of Increasing Utilization of Intravenous tPA for Acute Ischemic Stroke. Stroke 2005;36:2500-2503.

8. The ATLANTIS, ECASS, and NINDS rt-PA Study Group Investigators. Association of outcome with early stroke treatment: Pooled Analysis of ATLANTIS, ECASS, and NINDS rt-PA Stroke Trials. Lancet. 2004;363:768-74.

9. Adams HP, Effron MB, Torner J, Davalos A, et al for the AbESTT-II Investigators. Emergency Administration of Abciximab for Treatment of Patients with Acute Ischemic Stroke: Results of an International Phase III Trial: Abxicimab in Emergency Treatment of Stroke Trial (AbESTT-II). Stroke. 2008;39:87-99.

10. Johnston SC, Fung LH, Gillum LA, Smith WS, Brass LM, Lichtman JH, Brown AN. Utilization of intravenous tissue-type plasminogen activator for ischemic stroke at academic medical centers: the influence of ethnicity. Stroke. 2001;32: 1061-8.

11. Schumacher HC, Bateman BT, Boden-Albala B, Berman MF, Mohr JP, Sacco RL, Pile-Spellman J. Use of thrombolysis in acute ischemic stroke: analysis of the Nationwide Inpatient Sample 1999 to 2004. Ann Emerg Med. 2007; 50: 99-107.

12. Barber PA, Zhang J, Demchuk AM, Hill MD, Buchan AM. Why are stroke patients excluded from TPA therapy? An analysis of patient eligibility. Neurology. 2001;56: 1015-20.

13. Kelly-Hayes M, Wolf PA, Kase CS, Brand FN, McGuirk JM, D'Agostino RB (1995). Temporal patterns of stroke onset. The Framingham Study. Stroke. 1995*;*26:1343-7.

14. Magnusson S, Peterson TE, Sottrup‑Jensen L, Claeys H. Complete primary structure of prothrombin: isolation, structure and reactivity of ten carboxylated glutamic acid residues and regulation of prothrombin activation by thrombin. In: Reich E, Rifkin DB, Shaw E, editors. Proteases and biological control. New York: Cold Spring Harbor Laboratory, 1975:123–49.

15.Sottrup‑Jensen L, Claeys H, Zajdel M, Petersen TE, Magnusson S. The primary structure of human plasminogen: isolation of two lysine‑binding fragments and one “mini”‑plasminogen (MW, 38,000) by elastase‑catalyzed–specific limited proteolysis. In: Davidson JF, Rowan RM, Samama MM, Desnoyers PC, editors. Progress in chemical fibrinolysis and thrombolysis. New York: Raven Press, 1978:191–209.

16. Adams HP, del Zoppo G, Alberts MJ, et al. Guidelines for the Early Management of Adults With Ischemic Stroke. Stroke. 2007;38:1655-1711.

17. Hacke W, Kaste M, Bluhmki E, et al. Thrombolysis with Alteplase 3 to 4.5 Hours after Acute Ischemic Stroke. N Engl J Med 2008;359:1317-29.

APPENDIX A

Study Flowchart

*Include a study flowchart for ease of review and understanding of the study.*

| Evaluation | Screening  (Day 1) | Entry  (Day1) | 24 hrs | 36  hrs | 90 days |
| --- | --- | --- | --- | --- | --- |
| Informed Consent |  | X |  |  |  |
| Documentation of Disease/Disorder | X |  |  |  |  |
| Medical/Treatment History | X |  |  |  |  |
| Adverse Event Ascertainment |  | X | X | X | X |
| Clinical Assessment | X | X | X | X | X |
| Hematology | X |  |  |  |  |
| Chemistry | X |  |  |  |  |
| Non-Contrast Head CT or MRI | X | X | X |  |  |
| Pregnancy Testing | X |  |  |  |  |
| Questionnaires |  |  |  |  | X |

**APPENDIX B**


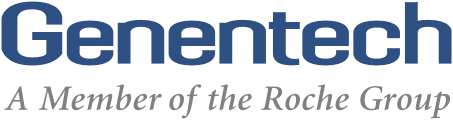
**SAFETY REPORTING FAX COVER SHEET**

##### **Genentech Supported Research**

AE / SAE FAX No: (650) 225-4682

Alternate Fax No: (650) 225-5288

| Genentech Study Number |  |
| --- | --- |
| Principal Investigator |  |
| Site Name |  |
| Reporter name |  |
| Reporter Telephone # |  |
| Reporter Fax # |  |

| Initial Report Date | [DD] / [MON] / [YY] |
| --- | --- |
| Follow-up Report Date | [DD] / [MON] / [YY] |

| Subject Initials  (Enter a dash if patient has no middle name) | [ ] - [ ] - [ ] |
| --- | --- |

SAE or Safety Reporting questions, contact Genentech Safety: (888) 835-2555

PLEASE PLACE MEDWATCH REPORT or SAFETY REPORT BEHIND THIS COVER SHEET

**APPENDIX C**

**Analysis of Similar Events Template for IND Safety Reports**

**IND Safety Report**

**Case Summary**

*This section will be initiated by a research coordinator and may be modified by principal investigators necessary. The case summary should describe the reported AE in detail, including a description of what happened and a summary of all relevant clinical information (e.g. medical status prior to the event, signs, symptoms, diagnoses, clinical course, treatment, outcome, etc.) The IND safety report should not identify the subject ID #, reporting investigator, or the site as this information may compromise the study blind.*

**PREVIOUS REPORTS**

*The information for this section comes from Principal Investigator and the search of similar events. This section should be written by the responsible principal investigator.*

** Select one of the following two statements after reviewing the search of similar events results.*

Under IND _______(insert IND#), the following IND safety reports of similar AEs have been previously submitted:

| MCN | Reported Event | Submission Date |
| --- | --- | --- |
|  |  |  |
|  |  |  |
|  |  |  |

Or

Under IND _______ (insert IND#), no IND safety reports of similar AEs have been submitted previously.

*In addition to previously submitted IND safety reports of similar events, this section can also summarize pervious serious reports of the same/similar event that were considered unrelated to the investigational product at the time of the reporting. These events would remain blinded, unless a decision to unblind is made by an Independent Monitoring Committee for reasons of subject protection. The decision on what similar events to summarize in this section should be made after reviewing the similar events report generated by Clinical Data Management. If a safety signal is particularly worrisome (e.g., a study stopping type of event), a more extensive evaluation may be required.*

**Assessment of Relationship**

*After evaluation the new case report and reviewing any relevant previous reports of similar events, the PI selects one of the following boilerplate conclusion statements, if applicable. The PI may also craft an alternative conclusion.*

Based on review of available data, (Insert Institution Name) believes there is a reasonable possibility of a cause-and-effect relationship between administration of _____________(insert study drug name) and the occurrence of _____________( insert AE).

*Additional information on risk factors and/or treatment of the AE may be provided if warranted.*

Or

Based on review of available data, the (Insert Institution Name) does not believe that there is a reasonable possibility of a cause-and-effect relationship between administration of _______(insert study drug name) and the occurrence of ___________(insert AE).

*Explain if warranted. Do not speculate.*

Or

Based on review of available data, the (Insert Institution Name) cannot establish or exclude the possibility of a cause-and-effect relationship between administration of __________(insert study drug name) and the occurrence of __________(insert AE).

*Explain if warranted. Do not speculate*.

After review of the clinical details and investigator’s comments pertaining to this AE, and based on experience to date, the (Insert Institution Name) does not believe that changes to the conduct of this clinical trial are warranted. *This statement can be modified if changes to the conduct of the clinical trial are made*.

Investigators participating in studies of Activase or Cathflo Activase should promptly submit this information to the Institutional Review Board or Independent Ethics Committee at their clinical site, and append this report to the Activase Investigator Brochure.

**APPENDIX D**

**Informed Consent**
